# Genome dynamics and chromosome structural variations in *Histoplasma ohiense*, a fungal pathogen of humans

**DOI:** 10.1093/g3journal/jkag118

**Published:** 2026-05-04

**Authors:** Sarah Heater, Mark Voorhies, Anita Sil

**Affiliations:** Department of Microbiology and Immunology, University of California SanFrancisco, San Francisco, CA 94143-0414, United States; Department of Microbiology and Immunology, University of California SanFrancisco, San Francisco, CA 94143-0414, United States; Department of Microbiology and Immunology, University of California SanFrancisco, San Francisco, CA 94143-0414, United States; Biohub, SanFrancisco, CA 94158, United States

**Keywords:** genome translocation, genome stability, genome assembly, mutation rate, *Histoplasma*

## Abstract

*Histoplasma* is a clinically important but understudied genus of thermally dimorphic human fungal pathogens. *Histoplasma* species normally transition between a multicellular sporulating hyphal form in the soil and a unicellular pathogenic yeast form in a mammalian host. Little is known about genome plasticity of *Histoplasma*, which we address in this study with the ultimate goal of increasing our understanding of its pathogenicity. Here we present the first telomere-to-telomere genome assemblies for *Histoplasma ohiense*; specifically, strains UCSF2 and UCSF3 derived from isolate G217B. We find that our 2 new assemblies differ from each other and from the previously published G217B reference genome by 2 reciprocal chromosome translocations. Analysis of short read sequencing of natural *Histoplasma* isolates reveals that the majority of isolates (91 of 94) match the chromosome structure of our UCSF3 assembly, which is, therefore, most representative of the original G217B isolate and *H. ohiense* in nature. Additionally, to determine the rate of *Histoplasma* genomic changes, we sequence 46 passaged isolates and calculate the mutation rate to be 2.6 × 10^−10^ SNP/base/doubling—the first such measurement to our knowledge within the order Onygenales, which encompasses several critical fungal pathogens. Taken together, this work highlights the plasticity of the *Histoplasma* genome and presents a comprehensive genome assembly that is representative of *H. ohiense* natural isolates.

## Introduction

The order Onygenales contains multiple understudied human fungal pathogens, several of which, including *Histoplasma* spp., rank as priorities on the WHO fungal pathogens list (WHO fungal priority pathogens list to guide research, development and public health action). Species in the genus *Histoplasma* are found globally, including endemic regions of high incidence in Africa and the Americas, with lifetime exposure rates thought to be over 80% in certain hyperendemic regions (Manos et al. 1956; Edwards et al. 1969; Moreira et al. 2022; WHO fungal priority pathogens list to guide research, development and public health action). *Histoplasma* infections occur when fungal cells are inhaled from the environment, where *Histoplasma* grows in a sporulating hyphal form. *Histoplasma* is thermally dimorphic and, upon inhalation, it has the remarkable capacity to transition to growth as yeast in response to mammalian body temperature (Sil 2019). Although *Histoplasma* can cause life-threatening infections even in healthy adults, severe Histoplasmosis infections are particularly common among immunocompromised individuals, with mortality estimates ranging up to 53% in HIV patients with disseminated histoplasmosis (Oladele et al. 2018; Sil 2019; Nacher et al. 2020; WHO fungal priority pathogens list to guide research, development and public health action). Treatment options for *Histoplasma* and other Onygenales are insufficient, and vaccines are not yet available (Oladele et al. 2018; Sil 2019; Nacher et al. 2020; WHO fungal priority pathogens list to guide research, development and public health action).

While *Histoplasma* has been found on all continents, the global incidence of *Histoplasma* is unknown (Moreira et al. 2022; WHO fungal priority pathogens list to guide research, development and public health action). Phylogenetic relationships from sequencing a set of DNA regions suggest at least 8 distinct clades of *Histoplasma* globally (Kasuga et al. 2003; Teixeira et al. 2016). A few recent studies have performed whole genome sequencing of *Histoplasma* clinical isolates primarily from North America, enabling a more detailed inference of population structure and sequence diversity in this region (Sepúlveda et al. 2017; Bagal et al. 2023; Tenório et al. 2024). Based on this sequencing, 4 *Histoplasma* species (*H. capsulatum* sensu lato) have been proposed (Sepúlveda et al. 2017). In this study, we will refer to *Histoplasma mississippiense* (also known as NAm1, reference genome derived from clinical isolate WU24) and *Histoplasma ohiense* (NAm2, reference genome derived from isolate G217B) as names for the 2 proposed species for which the largest amount of whole genome sequencing data are currently available.

We recently published the first 5 Nanopore-based *Histoplasma* genome assemblies. In this study, we find that our published *H. ohiense* reference genome contains structural differences from most *H. ohiense* clinical isolates and laboratory strains. We develop a more representative *H. ohiense* reference genome and in doing so find indications that *Histoplasma* may have a surprisingly high genome rearrangement rate. Additionally, we infer the single-nucleotide polymorphism (SNP) mutation rate. Mutation rate is particularly important for pathogens in the context of acquired treatment resistance, and we here make the first measurement of mutation rate in the order Onygenales and the second to our knowledge among Pezizomycotina (Álvarez-Escribano et al. 2019).

## Materials and methods

### Histoplasma strains

Rodriguez et al. 2019UCSF1, UCSF2, and UCSF3 are each minimally passaged strains of *H. ohiense* clinical isolate G217B. Previously sequenced natural isolates of *Histoplasma* used as reference include isolates defined as *H. ohiense* (or as NAm2) in 3 studies that previously published *Histoplasma* clinical isolate sequences: Bagal et al., Tenório et al., and Sepúlveda et al. (Sepúlveda et al. 2017; Bagal et al. 2023; Tenório et al. 2024). Strains used to define mutation rate were from the *Histoplasma* G217B *ura5Δ* (WU15) background. Strains used in this paper can be found in Supplementary Table 1.

### *Histoplasma* general growth conditions, DNA extraction, and sequencing

*Histoplasma* growth conditions were as described previously unless otherwise indicated (Assa et al. 2025). Extraction of genomic DNA for Illumina short read sequencing was performed as described previously (Assa et al. 2025). For all DNA extractions for short read sequencing, in any instance where a visible DNA precipitate did not appear after the addition of isopropanol, to ensure ample DNA was extracted, 1.5 μl GlycoBlue Coprecipitant (Invitrogen AM9515) was added, followed by an overnight incubation at 4 °C. Subsequent centrifugation steps were done at 4 °C instead of at ambient temperature. Illumina library preparation and sequencing was either performed using a Nextera DNA Flex Library Prep kit followed by sequencing at the UCSF Center for Advanced Technology or performed by SeqCenter, LLC (Pittsburgh, PA).

For the extraction of genomic DNA to be used for long-read Nanopore sequencing and associated short-read sequencing of these strains, 2 to 10 ml of a 3-day liquid culture was pelleted (2,500 rpm, 5 min), washed once in TE buffer (spin at 13,000 rpm for 1 min), and then stored at −80 °C until DNA extraction. DNA extraction was then performed using components of the Qiagen Gentra Puregene Yeast/Bacteria kit (158567) with a slightly modified protocol. Wide-mouth pipette tips were used when pipetting cell lysate or DNA, and vortexing was avoided other than as noted. *Histoplasma* cell pellets were resuspended in 300 μl cell suspension buffer, approximately 100 μl 0.5 mm glass beads were added, the mixture was thrice vortexed for 10 s, and put on ice for 10 s between each vortex. 4 μl of the lytic enzyme was then added; this mix was inverted 25 times, then incubated for 2 h at 37 °C with inversion 3 times an hour. This mixture was then pelleted at max speed for 1 min at 4 °C, supernatant was discarded, 300 μl cell lysis solution was added, then the mix was slowly pipetted several times and incubated at ambient temperature for 10 to 20 min. 100 μl protein precipitation solution was then added, the mixture was immediately vortexed for 5 s, placed on ice, vortexed for 1 s, then placed on ice for 5 min. This mix was pelleted at max speed for 3 min at 4 °C. Supernatant was then transferred to a tube containing 300 μl isopropanol and inverted 50 times to mix. This sample was pelleted at max speed for 2 min at 4 °C. Supernatant was removed, leaving approximately 50 μl so as not to disturb the DNA pellet. 500 μl 70% ethanol wash was added, then DNA was pelleted at max speed for 1 min. Supernatant was fully removed, pellet was air dried for 15 min, then 70 μl DNA hydration solution was added along with 1.5 μl RNase A solution. This was gently mixed by slowly pipetting up and down 10 times, then incubated at 37 °C for 5 min, 65 °C for 10 min, then overnight at 4 °C to dissolve the pellet. Nanodrop and TapeStation (Agilent Genomic DNA reagents) were used to assess quality and concentration prior to library preparation and sequencing at SeqCenter, LLC.

### SeqCenter sequencing

Illumina sequencing libraries were prepared using the tagmentation-based and PCR-based Illumina DNA Prep kit and custom IDT 10 bp unique dual indices (UDI) with a target insert size of 280 bp. No additional DNA fragmentation or size selection steps were performed. Illumina sequencing was performed on an Illumina NovaSeq X Plus sequencer in one or more multiplexed shared-flow-cell runs, producing 2 151 bp paired-end reads. Demultiplexing, quality control, and adapter trimming were performed with bcl-convert1 (v4.2.4).

Nanopore sample libraries were prepared using the PCR-free Oxford Nanopore Technologies (ONT) Ligation Sequencing Kit (SQK-NBD114.24) with the NEBNext Companion Module (E7180L) to manufacturer's specifications. No additional DNA fragmentation or size selection was performed. Nanopore sequencing was performed on an Oxford Nanopore GridION sequencer using R10.4.1 flow cells in one or more multiplexed shared-flow-cell runs. Run design utilized the 400 bps sequencing mode with a minimum read length of 200 bp. Adaptive sampling was not enabled. Guppy 1 (v6.5.7) was used for superaccurate basecalling, demultiplexing, and adapter removal (dna_r10.4.1_e8.2_400bps_modbases_5mc_cg_sup.cfg).

### Genome assembly and annotation

For each isolate (UCSF2 or UCSF3), reads were independently assembled by FLYE (Kolmogorov et al. 2019) version 2.9.4-b1799 with parameters –asm-coverage 40 –genome-size 40 m –nano-hq flags and CANU (Koren et al. 2017) v2.3-development (git commit c61ebbb7) with parameters genomeSize = 40 m and -nanopore. For both isolates, the FLYE and CANU assemblies were highly congruent, as assessed by NUCMER alignments (using NUCMER from MUMMER 3.23; Kurtz et al. 2004). The CANU assemblies were more complete with respect to telomeres and were used as the basis for manual finishing.

Manual finishing was accomplished by the general strategy of identifying highly identical ends between 2 contigs, trimming these ends to remove redundant overlap, and concatenating the trimmed contigs to generate a gapless join. We refer to this procedure below as “manually joining” and to the resulting contig–contig junctions as “manual joins” or simply “joins.” The full set of 4 manual joins is plotted in Supplementary Fig. 4 with supporting Nanopore long read alignments and annotation indicating the short-range repetitive nature of these regions.

The UCSF2 CANU assembly had 7 large contigs with 6 having telomeres on both ends and one (contig 4) having a telomere on only one end. A small contig with the final UCSF2 telomere was identified by NUCMER alignment of the UCSF2 CANU assembly to the UCSF3 FLYE assembly, and this small contig was joined to UCSF2 contig 4 based on the overlapping bases at the ends of the 2 contigs.

The UCSF3 CANU assembly had 9 large contigs, with 3 having telomeres on both ends and 5 (contigs 2, 3, 6, 8, and 9) having a telomere on only one end. A join between contigs 6 and 8 was identified by NUCMER alignment of the UCSF3 FLYE and CANU assemblies. A join between contigs 3 and 9 was identified by NUCMER alignment of the UCSF2 and UCSF3 CANU assemblies. Finally, we observed that contig 2 of the CANU assembly of UCSF3 ended in the 45S rDNA repeat, whereas the syntenic region in the UCSF2 assembly ended in the 45S rDNA repeat immediately followed by a telomere repeat with no intervening sequence. Therefore, we concatenated a telomere repeat to the 45S rDNA repeat of UCSF3 contig 2. After PILON version 1.23 (Walker et al. 2014) polishing with UCSF3 Illumina reads (see method below), we validated this extension by aligning UCSF3 Nanopore reads to the PILON polished assembly. Indeed, this identified 2 reads longer than 50 kb that include the 45S rDNA repeat followed by a telomere repeat with no intervening sequence (Supplementary Fig. 4d).

For both isolates, CANU assembled many copies of the mitochondrial DNA (mtDNA) as concatemers of different copy numbers. These mtDNA contigs were identified by alignment to the known mtDNA of the Sanger assembly of *H. capsulatum* G186AR (GCF_000150115.1 supercont1.87). For each isolate, a single mtDNA contig was selected and was clipped to single copy and circularized based on self comparison with NUCMER. For both isolates, after manual finishing of telomere-to-telomere nuclear chromosomes and the mtDNA, the remaining CANU contigs contained no unique sequence and were discarded.

Strand assignments were chosen to put syntenic chromosomes from UCSF2 and UCSF3 in matching orientations, and the assemblies were polished with paired-end Illumina reads from the corresponding isolate by up to 10 iterations of BWA MEM (Li 2013) version 0.7.17 and PILON version 1.23. Finally, the remaining Nanopore adapter sequence was identified and removed by trimming each nuclear chromosome to its terminal telomere repeats.

Final assemblies were annotated as in Voorhies et al. (2022): rRNA was annotated with RNAMMER 1.2 (Lagesen et al. 2007) in eukaryote mode, tRNA was annotated with TRNASCANSE 2.0.7 (Pavesi et al. 1994), LTR transposons were annotated with LTRHARVEST from GENOMETOOLS 1.6.1 (Ellinghaus et al. 2008) and TBLASTN from NCBI BLAST+ 2.11 (Altschul et al. 1997), and mRNAs were mapped from Gilmore et al. (2015) using BLAT v35 (Kent 2002) followed by repair of disrupted reading frames using TBLASTN and MEGABLAST from NCBI BLAST+ 2.11. Coordinates for these transposons are given in Supplementary Tables 4 to 7. As before, transposon-rich regions, which we refer to as LTR blocks, were defined by joining transposons within 50 kb of each other (Voorhies et al. 2022). Assembly statistics and completeness were assessed with BUSCO version 6.0.0 (Tegenfeldt et al. 2025) using the euriotomycetes_odb12 and ajellomycetaceae_odb12 ortholog sets, and the results are given in Supplementary Table 3.

### Analysis of structural variants

**Fig. 1.**
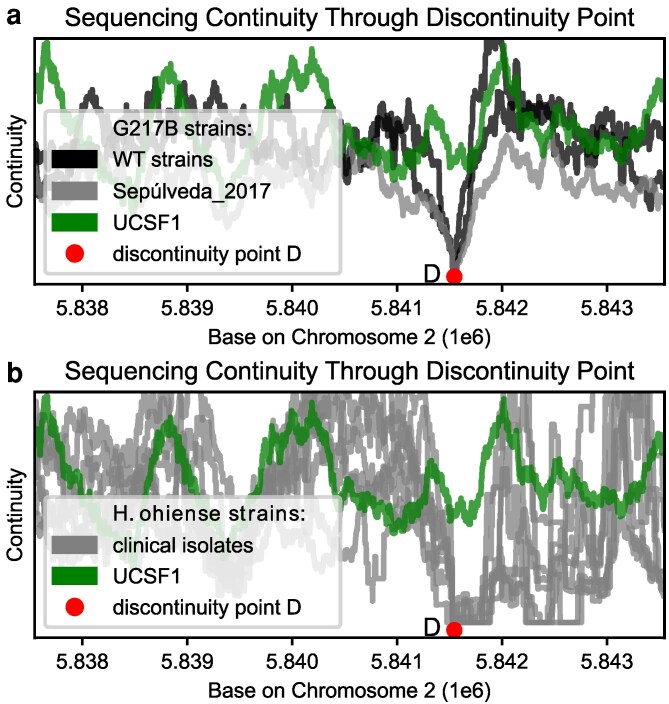
*Histoplasma ohiense* isolates have a consistent discontinuity with respect to the reference genome assembly. a, b) Continuity of Illumina reads aligned to the UCSF1 genome assembly for representative laboratory strains derived from clinical isolate G217B (a) and for representative clinical isolates (b). The strain from which the UCSF1 assembly was generated is shown in green, and the point of discontinuity for all other strains, D, is indicated with a red circle. Continuity was calculated as the read coverage spanning a given point relative to the read coverage in the vicinity of that point (see methods for details).

**Fig. 2.**
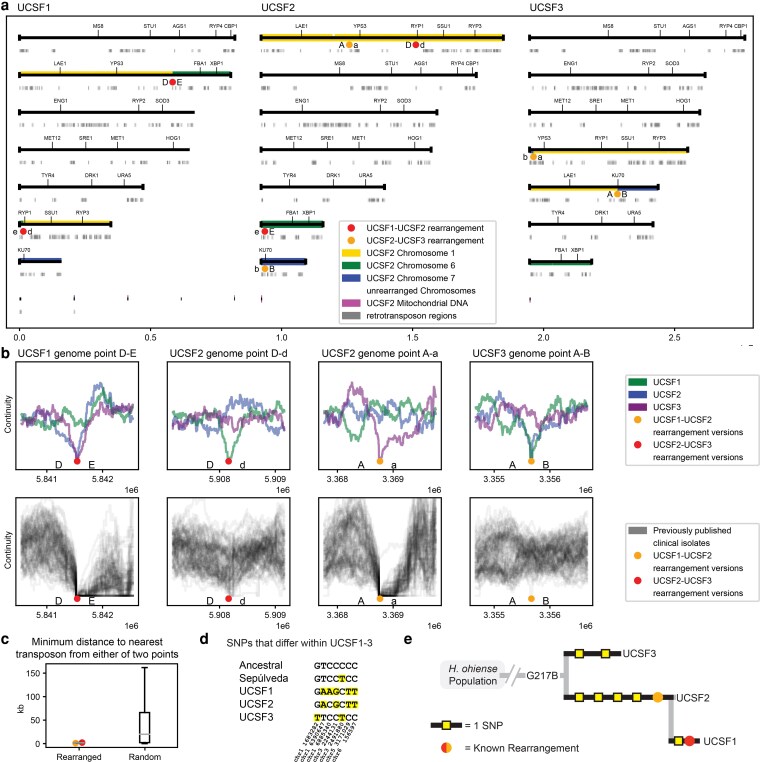
UCSF3 is the assembly most representative of laboratory and natural isolates. Two new genome assemblies were created from alternative isolates of *H. ohiense* G217B. a) Chromosome maps for the 3 G217B assemblies. Telomeres, if present, are indicated by black vertical lines at the end of each chromosome. The 3 chromosomes that contain rearrangements between assemblies are highlighted in yellow, green, and blue. NUCMER coordinates were used to color these chromosomes based on synteny to UCSF2. The mitochondrial DNA is shown in purple. Rearrangement breakpoints between UCSF1 and UCSF2 genomes are noted with red dots, while the rearrangements between UCSF2 and UCSF3 are noted with orange dots. Genes of interest are displayed on each chromosome as landmarks. LTR transposon regions are shown by grey blocks below the chromosomes. b) Continuity of Illumina read coverage at alternative versions of rearrangement breakpoints. The Illumina reads of the 3 strains from which the Nanopore assemblies were created are plotted at the top. Below in grey are continuity plots for previously sequenced *H. ohiense* natural isolates. c) Distance between rearrangement points and nearest LTR transposon versus other non-LTR-transposon points (10,000 random sets of 2 nontransposon points) with rearrangement points colored as in (a) and (b). d) Alleles for SNPs varying among UCSF1, UCSF2, and UCSF3. Genomic coordinates of each SNP are shown below the SNP matrix. Alleles for ancestral G217B (inferred by comparison to natural isolates of *H. ohiense*) and for a previously published sequence of a G217B isolate (Sepúlveda) (Sepúlveda et al. 2017) are included for comparison. e) Lineage tree of Nanopore-sequenced G217B-derived strains inferred by maximum parsimony from the SNP matrix (d) with SNPs and rearrangements acquired on each branch indicated by squares and circles, respectively.

We used DELLY 0.8.7 (Rausch et al. 2012) to infer structural variants (SVs) from our paired-end Illumina sequencing of the G217B-derived strains UCSF1, UCSF2, and UCSF3 and from previously published paired-end Illumina sequencing of 94 *H. ohiense* natural isolates. Specifically, we ran DELLY CALL on reads aligned to the UCSF1 reference with BWA MEM. In order to identify nontransposon rearrangements, we filtered the DELLY output BCF for BND SVs with FILTER = PASS, where both rearrangement positions were on nuclear chromosomes and removed variants where either position was in an LTR transposon or within 100 kb of a chromosome end. We considered SVs from different strains equivalent if both rearrangement positions from one strain were within 1 kb of the positions in the other strain. Based on this definition, we removed as false positives SVs detected by DELLY from UCSF1 Illumina reads aligned to the UCSF1 reference. The remaining SVs were clustered into equivalent rearrangements, using the above definition, and sorted by frequency across the 94 natural isolates. The two most common SVs (occurring in 93 or 92 strains, respectively) are reported in Figs. 1 and 2.

### Continuity of Illumina read coverage

The full method for the continuity metric plotted in Figs. 1 and 2b is given in supplemental code. In short, continuity was defined for each sequenced sample at each point in a reference genome as the number of read pairs overlapping the point divided by the number of read pairs that were aligned within 1 kb of the tested point. The 20 bp on each end of an aligned read pair are excluded to allow the index to consistently go fully to zero even in cases where short similarities are present on either side of the rearrangement. Read pairs with extreme separation between mates (over 100 kb or under 50 bp) are likewise excluded from the analysis. If no read pairs are aligned within 1 kb of a point (ie the numerator and denominator would both be 0), the output is instead set to −0.1.

### Visualization of syntenic regions among assemblies

Pairwise sequence alignments among the 3 G217B assemblies were generated using NUCMER with default parameters, and alignment coordinates were extracted from the resulting delta files using show-coords -r -c -l. These alignments are plotted in Supplementary Fig. 1 as lines joining the start and end coordinates in each genome. For Fig. 2a, the coordinates of the 1,000 longest alignments to UCSF2 chromosomes 1, 6, and 7 were plotted relative to each genome and colored by UCSF2 chromosome. In cases where a genomic region was included in multiple alignments, the longest alignment is shown. For the synteny visualization among distinct *Histoplasma* species in Supplementary Fig. 2, ortholog assignments were taken from Voorhies et al. (2022) and are reproduced in Supplementary Table 2.

### Analysis of retrotransposon locations and of distance to nearest retrotransposon region

For distance analysis for each translocation, the nearest LTR transposon locus was found on either side of the original pretranslocation points in the UCSF3 assembly. For comparison, the nearest repeat locus was also determined for each of 10,000 random sets of 2 nonrepeat loci.

### Inference of UCSF1/UCSF2/UCSF3 lineages relative to G217B

Paired-end Illumina reads from UCSF1, UCSF2, UCSF3, and 93 non-G217B *H. ohiense* population isolates were aligned to the UCSF2 reference using BWA MEM 0.7.17 with default parameters. SNP analysis was then carried out using the scripts and protocol of Voorhies et al. (2025) modified as follows: A SNP matrix was defined from the UCSF1, UCSF2, and UCSF3 read alignments as nucleotide positions covered by at least 10 reads from each isolate, with at least 85% of the reads from each isolate supporting a single allele, and with exactly 2 total alleles over all isolates. SNPs in LTR blocks, as defined above, were removed, leaving 10 SNPs. Allele ratios were then calculated for each of the 93 non-G217B read alignments for each SNP covered by at least 20 reads for at least 70% of the non-G217B samples, yielding allele ratios for 7 of the 10 original SNPs. At all 7 positions, the majority of reads for the majority of non-G217B samples unanimously assigned a single allele, which was taken as the inferred ancestral G217B allele, and a maximum parsimony tree was inferred for UCSF1, UCSF2, and UCSF3, rooted at the inferred G217B. This gave 4 SNPs shared by UCSF1 and UCSF2 on a common branch, 1 SNP unique to UCSF1, assigned to the branch following the divergence of UCSF1 and UCSF2, and 2 SNPs unique to UCSF3, assigned to an independent branch from the root.

### Assessment of mutation rate

Liquid cultures were started from single colonies, then passaged once to a second liquid culture. Cells in the passaged cultures were then counted by hemocytometer to calculate the total number of cells, which was used to calculate the number of doublings each parental cell had undergone. In order to account for the passaging step, either all cells from the previous culture were used, or hemocytometer counts were also taken at this intermediate stage to calculate doublings before and after this passage. After parental cultures were grown up, they were sonicated for 2 s, diluted, and offspring cells were plated for single colonies. Offspring colonies were allowed to grow until very small but clearly visible (about 1 wk) at 37 °C, then moved to 25 °C and allowed to grow for several weeks. Offspring colonies were then grown up via sequential patching on plates, followed by growth in liquid at 37 °C. Offspring genomic DNA was extracted and sequenced as described above. Two parental cultures were also sequenced to confirm our method of inferring parental sequences from offspring. Two previously sequenced strains were ancestral to all parental strains. In total, including data from 2 experiments, 46 offspring from 11 parental colonies were subjected to whole-genome sequencing for this analysis. There was an average of 31 doublings calculated from parental cells to offspring cells.

**Fig. 3.**
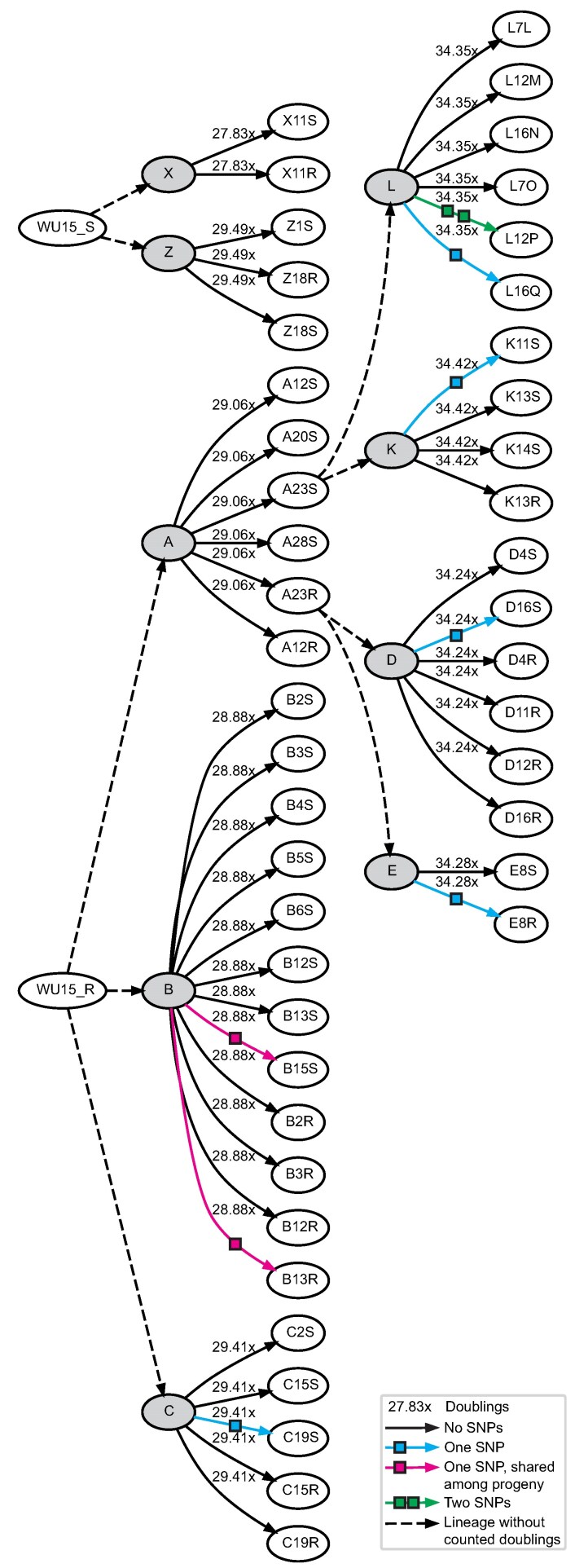
Schematic illustrating pedigree chart used to determine mutation rate. Relationships among clonal isolates (ellipses) are shown by arrows pointing from parents to offspring. Passaging without quantification is indicated by dashed arrows. Solid arrows indicate cases where hemocytometer counts were used to infer the number of doublings (numbers above arrows) between parents and offspring. Whole genome sequencing of offspring was used to infer SNPs (squares) due to mutation during passaging. The SNP rate was calculated as the total number of SNPs (squares) divided by the total number of doublings (summed doublings over all solid arrows) divided by the number of analyzed bases in the genome. See methods section for details.

Paired-end Illumina reads from the 46 offspring, 2 parental, and 2 ancestral samples were aligned to the UCSF3 reference using BWA MEM 0.7.17 with default parameters. A SNP matrix was defined from the read alignments as nucleotide positions covered by at least 10 reads from each isolate, with at least 85% of the reads from each isolate supporting a single allele, and with exactly 2 total alleles over all isolates. SNPs in LTR blocks, as defined above, were removed. Additionally, SNPs in chromosome 7, which is often duplicated, were removed so as to consider only haploid positions. The known relationships of the offspring relative to the sequenced and unsequenced parental strains were modeled as a tree, with branches labeled with number of doublings where available (Fig. 3). SNPs were then assigned to branches as follows: if all children of a given parent shared a SNP, then the SNP was assumed to arise at or before the parent; if exactly 2 children of a given parent differed by a SNP, then the SNP was assigned to the branch of the child that did not match the parent (1 case); if a child differed from all of its siblings by a SNP, then the SNP was assigned to the branch of that child (6 cases); finally, there was one case where the same SNP was observed in 2 out of 12 siblings. We tried handling this final case in 3 ways, all of which led to mutation rate estimates that were indistinguishable relative to their 95% confidence intervals (CIs): (i) the 2 identical SNPs were each included in calculations, assigned to the branches of both children; (ii) the SNP was assigned to one child's branch with the second child's branch dropped from the calculation; (iii) the SNP and the branches of both children were dropped from the calculation. These treatments gave a total of 9, 8, or 7 SNPs assigned to branches with measured doublings. The mutation rate was then calculated as the total number of assigned SNPs over the total doublings over all branches relative to the number of analyzed bases, and 95% CI was calculated from the binomial distribution using the binomtest function from SciPy stats (Zar 2010). For the above treatments, this gave: (i) 2.5873e−10 (95% CI 1.1834e−10 to 4.9122e−10), (ii) 2.3473e−10 (95% CI 1.0117e−10 to 4.6283e−10), or (iii) 2.0971e−10 (95% CI 8.4353e−11 to 4.3167e−10) SNPs per doubling per base.

### Additional software

Data were analyzed in JUPYTER 5.3.2 (Perez and Granger 2007) using PYTHON 3.12.3 with NUMPY 1.26.4 (van der Walt et al. 2011) and figures were generated using MATPLOTLIB 3.6.3 (Hunter 2007). Figure 3 was generated using NETWORKX 2.8.8 (Hagberg et al. 2008) and GRAPHVIZ 2.42.2 (Ellson et al. 2004).

## Results

### Analysis of genomic rearrangements in *H. ohiense*

From early studies of *Histoplasma* and recently published genome assemblies, it appears that chromosome size varies among clinical *Histoplasma* isolates from different species (Steele et al. 1989; Canteros et al. 2005; Voorhies et al. 2022). However, investigations of *Histoplasma* genome stability led us to the possibility that even very closely related *H. ohiense* strains might also have differences in chromosome composition. By mapping reads from lab strains derived from *H. ohiense* G217B onto the first Nanopore-based *H. ohiense* G217B assembly (Voorhies et al. 2022), we manually identified a point of discontinuity in read coverage on chromosome 2 of the reference assembly compared to all other tested lab isolates (Fig. 1a). Specifically, we originally identified this point (termed point D) because of a change in read coverage in some isolates (Heater et al. 2026) (ie a copy number variant boundary) and then realized that mapped reads did not span this point. Reads from most strains mapped partially to point D on chromosome 2 and partially to chromosome 6, suggesting that these regions were contiguous in many strains but not in the reference genome. These data suggested a chromosome rearrangement between sequenced strains and the closely related strain used to generate the reference genome. Previously sequenced clinical isolates of *H. ohiense* likewise exhibit a discontinuity at point D (Fig. 1b). We therefore hypothesized that the strain from which the previous assembly was generated did not have a representative chromosome structure compared to most *Histoplasma ohiense* isolates. Given this finding, we name the previous assembly of G217B “UCSF1” to clearly distinguish it from subsequent assemblies.

### Creation of a new reference sequence for *H. ohiense* and analysis of translocations

To generate an updated reference assembly for *H. ohiense*, we subjected 2 additional strains to Oxford Nanopore Technologies (ONT) sequencing as well as Illumina sequencing to generate the second and third *Histoplasma ohiense* genome assemblies, UCSF2 and UCSF3. All 3 strains used to generate *Histoplasma ohiense* reference genomes were derived from the same initial clinical isolate, G217B, the most extensively characterized isolate and the ancestral strain for all laboratory-generated *H. ohiense* mutants, including the widely used WU15 *ura5*^−^ strain. These 3 sequenced strains are only separated by minimal laboratory passage. In a first for *Histoplasma ohiense*, we were able to assemble complete telomere-to-telomere assemblies from both newly sequenced strains (Voorhies et al. 2022). As expected, these assemblies are highly syntenic (Fig. 2a, Supplementary 1 and 2). However, each *H. ohiense* assembly exhibits a slightly different chromosome structure. The region syntenic to the right-hand portion of chromosome 2 of UCSF1 (after point D) joins with a small telomeric section of chromosome 6 (at point e) to form a separate chromosome in both new assemblies; viz., chromosome 6 of UCSF2 and chromosome 7 of UCSF3 (Fig. 2a). We also detected an additional rearrangement in one of the new assemblies, UCSF3, relative to both other assemblies (corresponding to points A and B in UCSF2, Fig. 2a). As expected, Illumina reads from the strains used to generate both new assemblies did not have continuity through the previously described discontinuity point D when aligned to genome assembly UCSF1 (Fig. 2b).

The rearrangements among the 3 assemblies were validated by PCR of junction-specific primers on UCSF2 and UCSF3 genomic DNA (Supplementary Fig. 3, center column). As expected, primers targeting the UCSF1-specific junctions D/E and e/d gave no bands, while the corresponding junctions D/d and e/E gave positive bands for both UCSF2 and UCSF3. Likewise, A/a and b/B gave UCSF2-specific bands, and A/B gave a UCSF3-specific band. Primers targeting the UCSF3-specific b/a junction gave positive bands for both UCSF3 and UCSF2, likely due to the transposon-rich context of this junction, making it infeasible to uniquely target with short primers (Supplementary Fig. 3, middle left box). The strain-specific results for the 3 related junctions (Supplementary Fig. 3), and coverage of the b/a junction by Nanopore long reads (Supplementary Fig. 3, middle left box), lend confidence to the correct assembly of this junction in UCSF3.

To address which of these assemblies was most representative of the broader *H ohiense* population, we aligned Illumina reads from previously sequenced natural isolates (Sepúlveda et al. 2017; Bagal et al. 2023; Tenório et al. 2024) to the UCSF1 reference and used DELLY (Rausch et al. 2012) to find rearrangements among these isolates. The 2 most common rearrangements correspond to A/a and b/B of UCSF1 and UCSF2 rearranged to give b/a and A/B of UCSF3 (observed in 93 of 94 strains) and D/E and e/d of UCSF1 rearranged to give D/d and e/E of UCSF2 and UCSF3 (92 of 94 strains) (Fig. 2a). These results are consistent with continuity of read coverage at the points of rearrangement (Fig. 2b). We therefore concluded that assembly UCSF3 was most representative of the chromosome structure of natural *H. ohiense* isolates at these rearrangement points.

Genomic translocations in other fungi often occur near transposable elements (Fischer et al. 2000; Dunham et al. 2002; Umezu et al. 2002; Sui et al. 2020). We thus assessed the distance from each rearrangement point to the nearest identified LTR transposon. In keeping with the previously observed trends in other species, these translocation events were particularly close to transposon sequences, at 1.1 and 2.2 kb away versus a median of 19.6 kb for 10,000 randomly selected pairs of points (Fig. 2c).

To further assess how these strains relate to each other, as well as to other isolates within the *H. ohiense* population, we subjected UCSF1, UCSF2, and UCSF3 to SNP analysis. Mutations that differ between G217B-derived strains are likely to have arisen during laboratory passage and thus are not expected to be present in the environmental population. Throughout the environmental population, the nucleotide base at each of these loci should be consistent, and that base should reflect the content of the ancestral G217B isolate. We thus identified SNPs that differ amongst these 3 Nanopore-sequenced G217B-derived strains. To infer the most likely state of the common G217B ancestor, these SNPs were compared to those found in other natural isolates of *H. ohiense*. SNPs were also compared to a G217B-isolate-derived strain sequenced by a different group (Sepúlveda et al. 2017) (Fig. 2d). A maximum parsimony tree of this analysis, also showing the likely acquisition of identified genomic rearrangements, is shown in Fig. 2e. The tree inferred by SNP analysis was consistent with the tree inferred based on rearrangements, with UCSF3 showing the fewest SNPs relative to the inferred ancestral sequence. Because this assembly is the one most likely to resemble both the ancestral state of isolate G217B and *H. ohiense* in the wild, as measured by both SNPs and genome rearrangements, we suggest the use of assembly UCSF3 as the reference genome of *H. ohiense* in future genomic studies. We used this assembly for the remainder of this study.

We found it surprising that comparison of the 3 *H. ohiense* assemblies revealed 2 translocations and 7 SNPs (Fig. 2e). We are not aware of an analysis of the relative rates of SNP and translocations amongst fungi, especially through mitotic growth, but the mutation rate in the model fungus *Saccharomyces cerevisiae* is thought to be much higher than the rate of genomic translocations during mitotic growth (Sui et al. 2020). While we cannot retrospectively determine the number of doublings that separate the strains used to generate these *H. ohiense* assemblies, our findings suggested that during the laboratory passaging separating these isolates, the mutation rate might be unusually low or the translocation rate might be particularly high.

### First quantification of mutation rate in *Histoplasma*

The mutation rate in *Histoplasma* and in other fungi of the order *Onygenales* has not been previously measured. To assess SNPs per doubling, *H. ohiense* was grown up briefly in vitro, while cell doublings were counted, followed by identification of SNPs in offspring (Fig. 3). Doublings were monitored by counting cells with a hemocytometer as *H. ohiense* yeast was grown up first as single colonies, then briefly in liquid culture. Offspring were then grown up from single colonies and subjected to whole-genome Illumina sequencing. The sequences of “sibling” offspring were compared to each other to find mutations in particular offspring versus the offspring-consensus-inferred parental genome. Two parental genomes were also sequenced to spot check the accuracy of offspring-inferred parental genomes, confirming that we could correctly infer parental SNP content from concordance among offspring. As is standard for calculations of mutation rate, repetitive genomic regions that were highly enriched for transposons were excluded from mutation rate analyses due to ambiguous Illumina read mapping in these regions (Sui et al. 2020). We determined that the mutation rate in *H. ohiense* is 2.6 × 10^−10^ SNP/base/doubling (95% CI 1.2 × 10^−10^ to 4.9 × 10^−10^). This rate is similar to what is found in model yeast but slightly higher than observed in *Aspergillus*, the most closely related species for which a measured mutation rate is available (Álvarez-Escribano et al. 2019; Sui et al. 2020).

## Discussion

The ability to study *Histoplasma* genetics is dependent on a representative genome assembly. Our analysis of genome contiguity followed by de novo genome assembly of closely related *Histoplasma* isolates revealed that each assembled isolate contained a slightly different set of chromosomes. Chromosomal rearrangements affect the context in which genes lie and can affect gene expression, drive gene evolution, and are associated with fungal characteristics, including morphology, fitness, aneuploidy, and antifungal resistance (Tosato and Bruschi 2015). The initial impetus for our study was the identification of a genomic discontinuity defined by sequencing and analysis of natural and laboratory isolates. We were able to confirm this discontinuity via the generation of new Nanopore-based genome assemblies. By SV analysis of sequenced natural isolates and laboratory strains, we determined that the chromosome structure of *H. ohiense* UCSF3 was most representative of almost all sequenced strains. We subsequently found that UCSF3 is also most representative at the nucleotide level by SNP analysis.

In addition to being representative of *H. ohiense* isolates, UCSF3 also demonstrates the most synteny with assemblies of other *Histoplasma* species (Supplementary Fig. 2). Specifically, chromosome 5 of UCSF3 is syntenic to chromosome 4 of H88 (African clade), chromosome 6 of G186AR (*H. capsulatum*), and one side of chromosome 3 of WU24 (*H. mississippiense*), but this synteny is disrupted in UCSF1 and UCSF2 due to the rearrangement at point A/B producing a new chromosome (7 in both UCSF2 and UCSF1). Similarly, chromosome 7 of UCSF3 is syntenic to chromosomes 6 of UCSF2 and WU24 but is part of chromosome 2 of UCSF1 due to the rearrangement at point E/D and has undergone distinct rearrangements in G186AR and H88.

Aside from these differences, all of the *Histoplasma* assemblies show a high degree of synteny to UCSF3 chromosomes 1, 3, and 6, as we have noted previously (Voorhies et al. 2022), with chromosome 1 having a large inversion in *H. ohiense* relative to the other *Histoplasma* assemblies. Chromosome 3 of UCSF3 is notable for containing most genes involved in sulfur assimilation as well as the mating type locus (Fraser et al. 2007), and chromosome 6 of UCSF3 is notable for containing an approximately 2MB region centered on the morphological regulator DRK1 (Nemecek et al. 2006), which is also conserved in *Blastomyces*, *Paracoccidioides*, and *Coccidioides* (Voorhies et al. 2022).

We also assessed SNP rate and found that the mutation rate in *Histoplasma* is similar to mutation rates previously identified in other fungi and in other kingdoms of life. It remains to be seen if the rate might differ in hyphae or during morphological transitions, which is a technically challenging assessment since hyphal growth is by definition a multicellular form.

In assembling these genomes, we were surprised by the number of translocations observed among such closely related isolates, suggesting that during mitotic laboratory growth, the rate of *Histoplasma* translocations may be particularly high relative to model yeast. It is likely that a multitude of factors could influence translocation rates—for example, strong selective pressure during experimental evolution of fungi is associated with an increased rate of genomic translocation (Dunham et al. 2002), and it is possible that standard laboratory passage of *Histoplasma* triggers an unknown selection. However, even if the typical translocation rate is high through mitotic growth of *Histoplasma* in nature, cells with certain rearrangements may be selected against during meiosis, limiting the translocations among the environmental population and enabling the observed high degree of synteny between *Histoplasma* species. Alternatively, rearrangements may be primarily found in certain chromosomal regions, again enabling a high degree of synteny. A potential high mitotic translocation rate in *H. ohiense* could be associated with its high transposon content. Genomic rearrangements in other fungi are often adjacent to transposable elements (Dunham et al. 2002; Umezu et al. 2002; Sui et al. 2020), and *H. ohiense* genomes contain a high transposon content (20%) relative to other sequenced *Histoplasma* species and relative to model yeast (3%) (Kim et al. 1998; Voorhies et al. 2022). We find that rearrangement breakpoints among these 3 assemblies are on average closer to a transposon than average points within the genome, although the total count is too small for this to reach significance. In each of the identified translocations, one side of each rearrangement is found less than 2.5 kb from a transposon.

In *Schizosaccharomyces pombe*, genome-wide rearrangements with breakpoint junctions featuring microhomologies accumulate in quiescent, nondividing cells (Ellis et al. 2021). Similarly, Dunham et al. (2002) observed transposon-associated genome rearrangements in *S. cerevisiae* growing in nutrient-limiting conditions. They proposed that such rearrangements may be adaptive in yeast growing under strong selection and would be most likely to be observed in conditions favoring clonal growth, where cells would not be subject to the “generally catastrophic effects [of translocation] on fertility.” While the extent of nutrient deprivation and/or quiescence experienced by *Histoplasma* growing under standard passaging conditions is unclear, it is true that there is a delay in growth after cells are thawed from a frozen stock, and liquid cultures may enter stationary phase before being passaged to new media. Therefore, it is possible that starvation-induced mechanisms and/or selective pressure induced by nutrient limitation could be responsible for the rearrangements that we observe. *Histoplasma* does not recombine under normal laboratory conditions, as mating requires strains of opposite mating types as well as specific environmental conditions (Laskowski and Smulian 2010; Laskowski-Peak et al. 2012). Therefore, the high rate of rearrangements that we observe to occur during laboratory passage could likewise occur in yeast propagating clonally during mammalian infection, but would be expected not to persist in the natural population due to loss of fertility.

## Supplementary Material

jkag118_Supplementary_Data

## Data Availability

Annotated genome assemblies are available in GenBank as GCA_051312685.1 (UCSF2, PRJNA1196489) and GCA_051313235.1 (UCSF3, PRJNA1196486) with Illumina and Nanopore reads in SRA under the same BioProject accessions. Remaining sequencing reads are available in SRA under BioProjects PRJNA1256922 (Figs. 1 and 2) and PRJNA1257851 (Fig. 3). SRA records corresponding to specific strains are given in Supplementary Table 1. Systematic gene names and annotations for UCSF2 and UCSF3, and their correspondence to previously published *Histoplasma* gene annotations are given in Supplementary Table 2. Previously published sequencing of natural isolates from BioProjects PRJNA416769, PRJNA868688, and PRJNA1003095 was also used for analysis. Genome assemblies from BioProjects PRJNA682643, PRJNA682644, PRJNA682645, PRJNA682647, and PRJNA682648 were used for comparison. Code for read continuity analysis is also provided as a Supplementary file. Supplemental material available at G3 online.
