# Supplementary material for: Genome dynamics and chromosome structural variations in Histoplasma ohiense, a fungal pathogen of humans
Source: G3 (Bethesda). 2026 May 4;16(7):jkag118. doi: 10.1093/g3journal/jkag118 (PMC13334192; doi:10.1093/g3journal/jkag118)
Supplement: jkag118_Supplementary_Data [file jkag118_supplementary_data.zip › Supplemental_Material_Legends_G3-2026-406651.docx]

**Supplemental Figure 1. NUCMER Dotplot comparisons of the three *Histoplasma ohiense* genome assemblies**

**Supplemental Figure 2. Regions of synteny among UCSF2, UCSF3, and previously assembled *Histoplasma* genomes (Voorhies *et al*, 2022).**

Nuclear chromosomes for each genome are plotted as rectangles labeled by chromosome number. Orthologous genes (as annotated in supplemental table S2) are connected by lines colored by UCSF3 chromosome. Sites of rearrangement among the G217B derived strains (UCSF1, UCSF2, and UCSF3) are indicated by black vertical lines, labeled as in Fig. 2. Chromosomes are ordered and oriented to emphasize synteny.

**Supplemental Figure 3. PCR validation of rearrangements among UCSF1, UCSF2, and UCSF3.** PCR of diagnostic primers for the A/B rearrangement between UCSF3 and UCSF1/UCSF2 and the D/E rearrangement between UCSF2/UCSF3 and UCSF1. Center column shows agarose gels of PCR products for UCSF2 or UCSF3 gDNA templates with PCR targets labeled above gel lanes. PCR target labels are connected to plots showing features of each junction relative to chromosome coordinate (x-axis); top: minimap2 aligned Nanopore reads overlapping the junction breakpoint by at least 2kb on either side (blue, with unaligned soft-clipped portions of reads in green); middle: number of BLASTN hits vs. full genome for 100bp sequenced tiled across the plotted region, plotted on a log scale (black line); bottom: PCR target (green line), minimap2 aligned chromosome fragments (red and magenta lines) from genomes differing at rearrangement point (dashed vertical red line). Outermost plots indicate the genomic locations of the BLASTN hits (green blocks) relative to annotated transposons (gray blocks) and the site of rearrangement (red vertical bar). Chromosomes are arranged vertically largest to smallest as in Fig. 2.

**Supplemental Figure 4. Long read support for manual joins.** Read alignment and sequence details for the single manual join in the UCSF2 assembly (A) and the three manual joins in the UCSF3 assembly (B-D). A-C) minimap2 aligned Nanopore reads overlapping the right side of the join by at least 2k on either side (top frame) the left side of the join by at least 2kb on either side (second frame) or spanning the entire join (third frame). Aligned reads are plotted in blue with unaligned soft-clipped portions of reads in green. Telomere repeats in aligned reads are indicated as cyan vertical bars. Black lines (fourth frame) indicate number of BLASTN hits vs. full genome for 100bp sequenced tiled across the plotted region, plotted on a log scale. As in Fig. S3, these BLASTN hits correspond to annotated transposon regions. Bottom plots minimap2 alignment of joined contig ends from earlier assembly steps in red and magenta with dashed vertical lines indicating the corresponding boundaries of the join regions. Gapless joins with no scaffolding Ns were accomplished by merging contigs on the long overlapping identical subsequences. D) Manual join of UCSF3 45S rDNA to telomere repeat. Top 3 frames show all minimap2 aligned Nanopore reads (top frame), minimap2 aligned Nanopore reads with 3' telomere repeats (second frame), and join-spanning minimap2 aligned Nanopore reads greater than 10 kb (third frame). The bottom frame plots annotated lsu (dark gray) and ssu (light gray) rDNA and genomic 3' telomere repeats (vertical cyan bars). Red dashed vertical lines indicate the 3' ends of the 3 final ssu rDNA repeats and coincide with 3' telomere repeats in the aligned Nanopore reads (second frame), supporting direct continuity between the 3' end of the 45S rDNA and the telomere.

**Supplemental Table 1.** Strains used in this paper

**Supplemental Table 2.** Gene annotations among genome assemblies

**Supplemental Table 3.** Genome assembly statistics and BUSCO analysis.

**Supplemental Table 4.** UCSF2 transposon locations annotated by TBLASTN of gag and pol sequences in GFFv3 format.

**Supplemental Table 5.** UCSF2 transposon locations annotated by LTRharvest in GFFv3 format.

**Supplemental Table 6.** UCSF3 transposon locations annotated by TBLASTN of gag and pol sequences in GFFv3 format.

**Supplemental Table 7.** UCSF3 transposon locations annotated by LTRharvest in GFFv3 format.

**Supplemental Code.** Python implementation of the continuity calculations in Fig. 1 and Fig. 2B.
