# Supplementary figures and images for: Genome dynamics and chromosome structural variations in Histoplasma ohiense, a fungal pathogen of humans
Source: G3 (Bethesda). 2026 May 4;16(7):jkag118. doi: 10.1093/g3journal/jkag118 (PMC13334192; doi:10.1093/g3journal/jkag118)

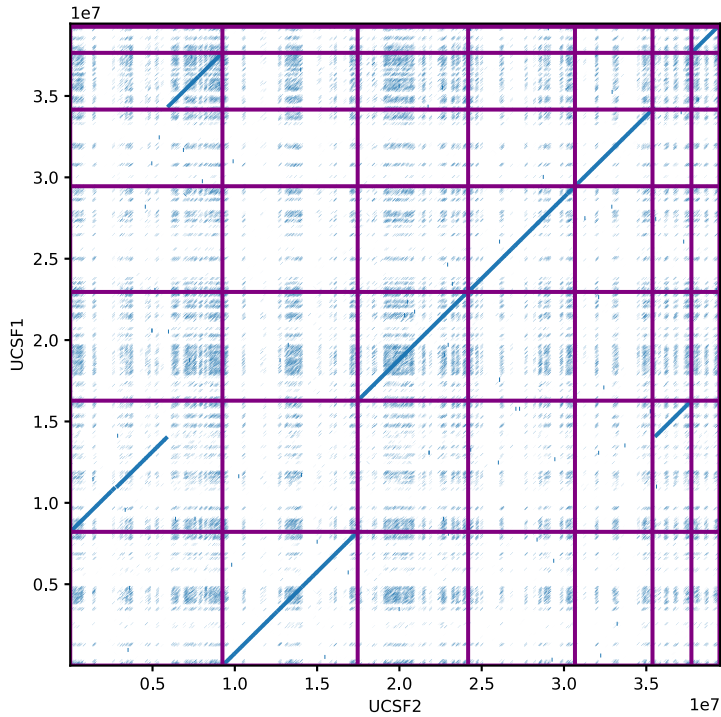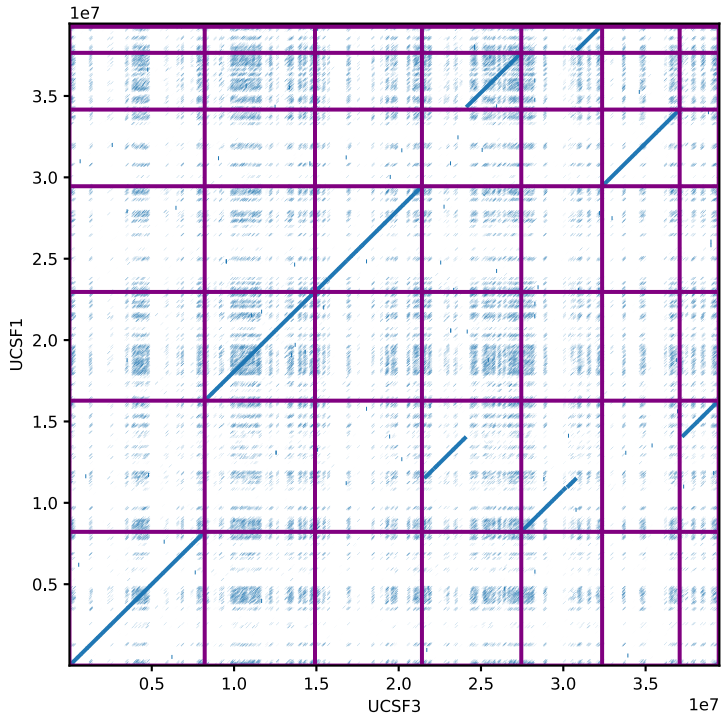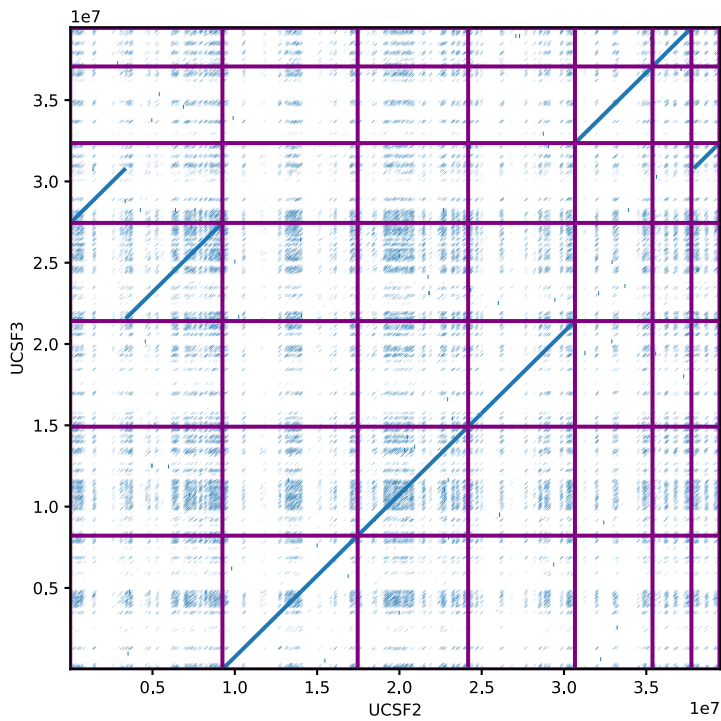

Supplement: jkag118_Supplementary_Data [file jkag118_supplementary_data.zip › Fig._S1_G3-2026-406651.pdf]
